# Harmonizing Motherhood: Navigating Pregnancy Outcomes in Connective Tissue Disorders

**DOI:** 10.7759/cureus.62134

**Published:** 2024-06-11

**Authors:** Manju Mathesan, Shanthi Ethirajan

**Affiliations:** 1 Obstetrics and Gynaecology, Saveetha Institute of Medical and Technical Sciences (Deemed to be University), Chennai, IND

**Keywords:** antiphospholipid antibodies, maternal-fetal health, fetal maternal outcomes, pregnancy, connective tissue disorder

## Abstract

Background

Connective tissue disorders encompass a diverse array of autoimmune and hereditary conditions, including systemic lupus erythematosus (SLE), rheumatoid arthritis (RA), and antiphospholipid antibody syndrome. These disorders present unique challenges during pregnancy due to their complex pathophysiology and potential complications. Understanding their impact on pregnancy outcomes is vital for optimizing maternal and fetal health.

Objective

To investigate the burden, complications, maternal and fetal outcomes, and prognosis of connective tissue disorders in pregnancy.

Methods

The study was conducted over one year and six months at Saveetha Medical College and Hospital, Chennai, India, involving 45 pregnant women diagnosed with connective tissue disorders. Standard antenatal investigations were conducted, and participants were monitored throughout the antenatal period. Maternal and fetal outcomes were meticulously evaluated.

Results

Baseline characteristics revealed a heterogeneous distribution of age and parity among participants, reflecting the diverse nature of connective tissue disorders in pregnancy. Maternal medical outcomes, such as gestational hypertension (GHTN) and gestational diabetes mellitus (GDM), were prevalent, highlighting the necessity of close monitoring. Obstetric outcomes included spontaneous abortion and preterm delivery, indicating elevated risks in this population. Fetal outcomes, including fetal growth restriction and admission to the neonatal intensive care unit, underscored the impact of these disorders on fetal health.

Conclusion

This study examines pregnant connective tissue disorder burden, complications, maternal and fetal outcomes, and prognosis. The complicated relationship between these illnesses, and pregnancy requires specialist care and close monitoring. The participants' baseline features represent connective tissue condition heterogeneity, affecting clinical practice. Among the study subjects, 40% had RA and 20% had SLE, the most common connective tissue illness. Adverse maternal medical outcomes, like GHTN (27.27% of antiphospholipid syndrome (APS) patients and 22.22% of SLE patients) and GDM (18.18% of APS patients and 11.11% of SLE patients), highlight the need for close maternal health monitoring and management during pregnancy. Overall, this study sheds light on connective tissue abnormalities and pregnancy outcomes. Healthcare providers can improve reproductive health and well-being for various illnesses by knowing these relationships.

## Introduction

Rogerius, a physician from the 13th century, coined the term "Lupus" to characterize erosive face lesions in systemic lupus erythematosus (SLE) that resembled a wolf's bite [1]. Connective tissue disorders can be classified into distinct groups: hereditary connective tissue disorders include Ehlers-Danlos syndrome, Marfan's syndrome, and pseudoxanthoma elasticum. Autoimmune connective tissue disorders, including SLE, antiphospholipid antibody (APLA) syndrome, scleroderma, and polymyositis, involve the body's immune system attacking its own tissues. Patients in this category generally have increased levels of antinuclear antibodies (ANAs) and anti-ribonucleoprotein (anti-RNP) antibodies. Classifying mixed connective tissue disorders (MCTDs) and overlap syndromes is challenging due to their combination of three separate diseases [2].

Connective tissue disorders include both autoimmune diseases like SLE and rheumatoid arthritis (RA), as well as genetic disorders like scleroderma [3]. These illnesses cause anomalies in connective tissues, affecting various organs and systems. Limited research has been conducted on the impact of these conditions on pregnancy outcomes, despite significant advances in understanding their clinical presentation and management [4]. Pregnancy poses unique physiological challenges, especially for those with connective tissue disorders who may have additional complications. The complicated interaction of pregnancy with connective tissue disorders, including altered immune response, inflammation, and hormonal alterations, highlights the need for further research into pregnancy outcomes in this population [3].

Understanding the unique challenges and dangers associated with pregnancy in people with connective tissue disorders is crucial for providing tailored and educated treatment advice. Understanding this concept can help healthcare workers improve prenatal care, efficiently handle difficulties, and make informed treatment decisions during pregnancy [5]. This study aims to investigate how connective tissue abnormalities affect a mother's health and fetal outcomes. Examining pregnancy outcomes can help identify the chances of adverse events such as preterm birth, hypertension, and fetal growth limitation in persons with these diseases [6].

Individuals with connective tissue disorders may require ongoing medical management, including immunosuppressive therapies [2]. This study evaluates the safety and effectiveness of therapies for connective tissue disorders during pregnancy, providing doctors with evidence-based data to guide pharmaceutical management decisions. Researching the impact of connective tissue disorders on pregnancy outcomes offers vital insights into long-term consequences for both mothers and their children. This understanding is crucial for designing measures to improve the health and well-being of people with connective tissue disorders, and their children, beyond the perinatal period [4].

This study investigates the link between connective tissue abnormalities and pregnancy outcomes. The discoveries could have a considerable impact on clinical practices and improve treatment. Developing solutions can improve reproductive health and overall well-being for those living with complex diseases. This study examines the impact of collagen vascular disorders on pregnancy and its results for both mothers and babies.

## Materials and methods

This prospective observational study was conducted in the Obstetrics and Gynaecology Department at Saveetha Medical College and Hospital, Chennai, India, over a duration of one year and six months, spanning from March 2022 to September 2023. With a sample size of 45 participants, the patients were recruited as shown in Table 1. The study included women with previously diagnosed connective tissue disorders and those newly diagnosed. Standard antenatal investigations, such as hemoglobin level, urine routine, blood grouping, Rh typing, glucose challenge test (GCT), serology, electrocardiogram, and echocardiography, were performed. Pregnant women with connective tissue disorders were actively followed during the antenatal period, and both maternal and fetal outcomes were thoroughly examined.

**Table 1:** Inclusion and exclusion criteria ANC: antenatal care

Inclusion criteria	Exclusion criteria
All pregnant women presenting with connective tissue disorders to ANC OPD or rheumatology OPD were included in this study	Patients not willing to participate in the study
Newly diagnosed	Patients lost to follow-up
Willing to participate in the study	Patients diagnosed with intrauterine death

## Results

Tables 2-3 outline the baseline characteristics of the study participants, categorized by their respective connective tissue disorders: APLA, SLE, RA, MCTD, and Sjogren’s syndrome. The parameters include age in years and parity status (primigravida or multigravida), with the number of participants and their respective percentages within each disorder group provided. For instance, in the age category of 20-25 years, within the antiphospholipid syndrome (APS) group, there were five participants, accounting for 45.45% (5) of the total APS participants. Similarly, within the SLE group, there were three participants in the same age category, representing 33.33% (3) of the SLE participants. This pattern continues for the other age groups and disorder categories. Additionally, under the parity parameter, the table indicates that within the study population, 66.67% (30) of participants with connective tissue disorder were primigravida (first-time pregnant), while 33.33% (15) were multigravida (having had multiple pregnancies).

**Table 2:** Baseline characteristics of the study participants SLE: systemic lupus erythematosus, MCTD: mixed connective tissue disorder; APLA: antiphospholipid antibody; RA: rheumatoid arthritis The statistical parameter in which the data has been represented in the table is N(%)

Parameter (age in years)	APLA syndrome (n = 11)	SLE (n = 9)	RA (n = 18)	MCTD (n = 5)	Sjogren’s syndrome (n = 2)
20-25	5 (45.45%)	3 (33.33%)	6 (33.33%)	2 (40%)	0
25-30	4 (36.36%)	4 (44.44%)	5 (27.77%)	3 (60%)	2 (100%)
30-35	2 (18.18%)	2 (22.22%)	7 (38.88%)	0 (0)	0

**Table 3:** Baseline characteristics of the study participants The statistical parameter in which the data has been represented in the table is N(%)

Parity	Number of patients N(%)
Primigravida	30 (66.67%)
Multigravida	15 (33.33%)

**Figure 1:**
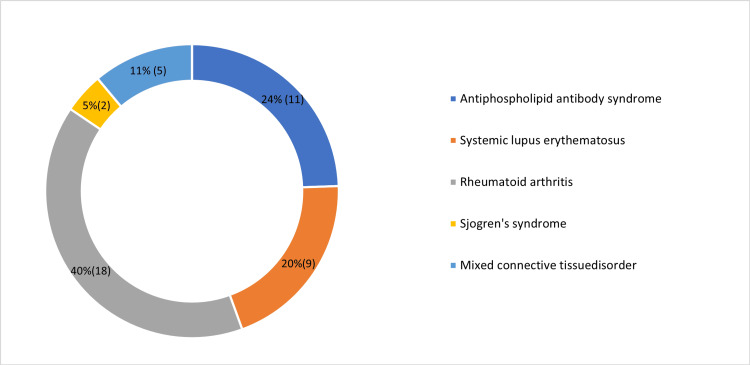
Distribution of the study participants The statistical parameter in which the data has been represented in the figure is %(N)

Of the 45 participants, 24% (11) had APLA, 40% (18) had RA, 20% (9) had SLE, 11% (5) had MCTD, and 5% (2) had Sjogren’s syndrome as shown in Figure 1.

Outcomes of the study participants

Table 4 highlights the outcomes of the study participants.

**Table 4:** Maternal, obstetric, and fetal outcomes in the study participants APLA: antiphospholipid antibody; RA: rheumatoid arthritis; HTN: hypertension; GDM: gestational diabetes mellitus; NVD: normal vaginal delivery; LSCS: lower segment cesarian section; FGR: fetal growth restriction; NICU: neonatal intensive care unit The statistical parameter in which the data has been represented in the table is N(%)

Outcome	APLA syndrome (n = 11)	SLE (n = 9)	RA (n = 18)	MCTD (n = 5)	Sjogren’s syndrome (n = 2)
Maternal medical					
Gestational HTN	3 (27.27%)	2 (22.22%)	2 (11.11%)	0 (0)	1 (50%)
Chronic HTN	1 (9.09%)	1 (11.11%)	2 (11.11%)	0 (0)	0 (0)
Renal disease	0 (0)	2 (22.22%)	0 (0)	0 (0)	0 (0)
Thrombocytopenia	0 (0)	0 (0)	2 (11.11%)	0 (0)	0 (0)
GDM	2 (18.18%)	1 (11.11%)	1 (5.55%)	2 (40%)	1 (50%)
None	4 (36.36%)	3 (33.33%)	11 (61.11%)	3 (60%)	0 (0)
Obstetric					
Spontaneous abortion	5 (45.45%)	6 (66.66%)	3 (16.66%)	0 (0)	0 (0)
Full term NVD	3 (27.27%)	1 (11.11%)	8 (44.44%)	0 (0)	1 (50%)
Pre-term NVD	0 (0)	0 (0)	2 (11.11%)	1 (20%)	0 (0)
Full term LSCS	3 (27.27%)	2 (22.22%)	5 (27.77%)	2 (40%)	1 (50%)
Pre-term NVD	0 (0)	0 (0)	0 (0)	0 (0)	0 (0)
Fetal					
Full term	6 (54.54%)	3 (33.33%)	10 (55.55%)	2 (40%)	1 (50%)
Pre-term	0 (0)	0 (0)	2 (11.11%)	1 (20%)	1 (50%)
FGR	2 (18.18%)	1 (11.11%)	0 (0)	0 (0)	0 (0)
NICU admission	3 (27.27%)	1 (11.11%)	3 (16.66%)	1 (20%)	0 (0)

Maternal Medical Outcomes

Parameters such as gestational hypertension (GHTN), chronic hypertension, renal disease, thrombocytopenia, and gestational diabetes mellitus (GDM) are assessed. Within the APS group, 27.27% (3) experienced GHTN, 9.09% (1) had chronic hypertension, and 18.18% (2) developed GDM.

Obstetric Outcomes

Obstetric outcomes include spontaneous abortion, full-term normal vaginal delivery (NVD), pre-term NVD, full-term lower segment cesarean section (LSCS), and pre-term delivery. In the RA group, 16.66% (3) experienced spontaneous abortion, 44.44% (8) had full-term NVD, and 11.11% (2) underwent pre-term NVD.

Fetal Outcomes

Fetal outcomes encompass full-term delivery, pre-term delivery, fetal growth restriction (FGR), and neonatal intensive care unit (NICU) admission. In the SLE group, 33.33% (3) of infants were born pre-term, 11.11% (1) experienced FGR, and 11.11% (1) required NICU admission.

## Discussion

Autoimmune illnesses pose distinct concerns during pregnancy due to their complex pathology and possible consequences. This study examined the prevalence, complications, maternal and fetal outcomes, and prognosis of connective tissue abnormalities in pregnancy. The study's participants' baseline features represent the diversity of connective tissue disorders during pregnancy, providing useful insights into their management and care. Understanding demographic characteristics is crucial for examining pregnancy outcomes, as shown by the distribution of age and parity across illnesses. Certain illnesses, such as RA and SLE, have considerable prevalence and clinical significance in obstetric practice.

Our study found a varied age range and parity among individuals, highlighting the pregnant connective tissue disorder burden, complications, maternal and fetal outcomes, and prognosis. Previous research by Pastore et al. [1] and Cervera et al. [2] found that pregnant individuals with connective tissue disorders have a diverse age range and parity status. Our analysis found similar prevalence rates for certain illnesses, as stated in the literature. Our study found a high prevalence of RA and SLE, which aligns with studies by Cervera et al. [2] and Braga et al. [3]. These disorders are clinically significant in obstetric practice.

Our study found changes in baseline parameters compared to earlier research. Our study included a somewhat different proportion of primigravida and multigravida persons compared to Louthrenoo et al. [6], who found a larger prevalence of primigravida among pregnant patients with connective tissue disorders. Variations in healthcare environments, access to prenatal treatment, and cultural variables may impact the demographics of pregnant individuals with connective tissue disorders in studies. The constancy of baseline features across research highlights the need to investigate the demographic and clinical profile of pregnant persons with connective tissue disorders. These insights are essential for personalizing prenatal care, adopting targeted interventions, and improving mother and fetal outcomes in this vulnerable population.

The study found maternal medical and obstetric outcomes linked to connective tissue diseases. The study found a high prevalence of GHTN, chronic hypertension, and GDM, emphasizing the importance of monitoring and managing maternal health during pregnancy. Individuals with these diseases are more likely to experience unfavorable pregnancy outcomes, including spontaneous abortion, premature delivery, and cesarean section. Fetal connective tissue abnormalities have a significant impact on fetal health, as evidenced by outcomes such as growth limitation and NICU hospitalization. Preterm delivery and fetal distress led to NICU admission, emphasizing the importance of fetal monitoring and timely intervention for better neonatal outcomes.

Understanding connective tissue disorders during pregnancy can inform prenatal treatment, interdisciplinary management approaches, risk assessment, and patient support [7]. Prenatal treatment procedures for pregnant individuals with connective tissue disorders should be tailored to their specific needs. A multidisciplinary approach comprising obstetricians, rheumatologists, and other experts ensures complete management and timely intervention. Early risk assessment and stratification can identify high-risk patients and prevent negative consequences [8]. Educating patients about their condition and providing emotional support promote active participation in their care [9].

Obstetricians face significant problems when diagnosing and treating autoimmune diseases during pregnancy. Pregnancy in individuals with autoimmune illnesses is high-risk and requires close monitoring to prevent complications for both the mother and the baby. Close fetal observation is critical to improving outcomes. Women with SLE often have increased disease activity during pregnancy. Among nine SLE patients in our study, 66% had spontaneous abortions. Molad et al. found a 20.7% spontaneous abortion rate among 29 pregnancies with SLE in their study [10].

Mankee et al. found that lupus anticoagulant positivity in the first trimester predicts pregnancy loss, accounting for 66% of spontaneous abortions in their study [11]. In our analysis of nine SLE patients, five had medical comorbidities. The most common was hypertensive disorders of pregnancy (33%), followed by lupus nephritis (22.2%). Our study found that 45% of patients with APLA syndrome had spontaneous abortions. Medical therapies like aspirin and heparin can improve pregnancy outcomes for those with recurrent miscarriages linked to APLAs. A meta-analysis found that taking aspirin with unfractionated heparin improves live birth rates and reduces miscarriage rates by 54% compared to using aspirin alone [12].

Future research directions include mechanistic investigations to understand underlying processes, longitudinal cohort studies to validate findings and assess long-term consequences, reviews of treatment strategies, and health outcomes research to explore larger implications. Research into molecular pathways linked to negative pregnancy outcomes, such as placental malfunction or immune-mediated fetal damage, could provide new insights into possible treatment targets [13]. Studying the impact of pro-inflammatory cytokines like tumor necrosis factor-alpha (TNF-α) and interleukin-6 (IL-6) on placental dysfunction and FGR in patients with connective tissue disorders could lead to targeted interventions [14,15]. Understanding the role of autoantibodies, such as anti-phospholipid antibodies, in pregnancy-related thrombotic events and vascular damage could lead to new prevention strategies [16]. Pregnant women with connective tissue disorders may experience immune modulation and disease activity due to hormonal changes, such as changes in estrogen and progesterone levels [17]. This could reveal mechanisms underlying disease exacerbation or remission. Mechanistic investigations can help create personalized treatments for connective tissue abnormalities during pregnancy, leading to better mother and fetal outcomes.

The study's weakness is that it was conducted at a single medical center with a sample size of 45 participants. The results may not be applicable to all pregnant individuals with connective tissue issues. Larger multicenter trials with diverse demographics and clinical variables may improve the generalizability of findings. The study relied on gathering data retrospectively for specific variables. This may lead to recollection bias or poor documentation, thereby compromising data accuracy and dependability. The study's conclusions may be limited by the availability and completeness of clinical data, including participants' medical backgrounds, illness severity, medication plans, and treatment adherence. Insufficient documentation and data can hinder understanding of how connective tissue disorders affect pregnancy outcomes.

The lack of a control group of pregnant individuals without connective tissue disorders hinders direct comparisons and understanding of the influence of these conditions on pregnancy outcomes. Including a control group allows researchers to pinpoint the precise influence of connective tissue abnormalities on poor maternal and fetal outcomes rather than other possible reasons.

## Conclusions

This study thoroughly examines the impact, complexities, and consequences of connective tissue abnormalities during pregnancy, focusing on the future outlook for both the mother and fetus. The findings emphasize the intricate interaction between these illnesses and pregnancy, highlighting the necessity for specialized care and careful monitoring. Participants displayed diverse connective tissue illnesses, significantly influencing therapeutic practice. RA and SLE were the most common conditions, accounting for 40% and 20% of the sample, respectively. The study noted significant negative maternal outcomes such as GHTN (27.27% in APS patients and 22.22% in SLE patients) and GDM (18.18% in APS patients and 11.11% in SLE patients), underscoring the importance of closely monitoring maternal health during pregnancy. Obstetric problems were highly common, with 66.66% of SLE patients and 45.45% of APS patients experiencing spontaneous abortion. Additionally, 33.33% of SLE cases resulted in premature delivery. A significant number of APS (27.27%) and SLE (22.22%) patients underwent full-term LSCS. Fetal outcomes were also affected, with FGR observed in 18.18% of APS patients, and NICU stays necessary for 27.27% of infants with APS and 11.11% with SLE. These findings emphasize the need for a multidisciplinary approach involving obstetricians, rheumatologists, and other specialists to ensure comprehensive management and timely interventions. Future research should focus on investigating the fundamental mechanisms behind adverse pregnancy outcomes linked to connective tissue disorders, validating current findings, evaluating long-term health outcomes, and assessing the effectiveness of various treatment approaches. This study's insights can help healthcare providers develop more effective strategies to improve reproductive health and well-being for those affected by these conditions.

## References

[1] Pastore DE, Costa ML, Surita FG (2019). Systemic lupus erythematosus and pregnancy: the challenge of improving antenatal care and outcomes. Lupus.

[2] Cervera R, Serrano R, Pons-Estel GJ (2015). Morbidity and mortality in the antiphospholipid syndrome during a 10-year period: a multicentre prospective study of 1000 patients. Ann Rheum Dis.

[3] Braga A, Barros T, Faria R (2021). Systemic lupus erythematosus and pregnancy: a retrospective single-center study of 215 pregnancies from Portugal. Lupus.

[4] Ruffatti A, Salvan E, Del Ross T (2014). Treatment strategies and pregnancy outcomes in antiphospholipid syndrome patients with thrombosis and triple antiphospholipid positivity. A European multicentre retrospective study. Thromb Haemost.

[5] Pajor A, Pozsonyi T, Nékám K, Bakos L, Haraszti L, Paulin F (1998). Systemic lupus erythematosus and pregnancy (effect of pre-conception hematologic disorders on fetal outcome) (Article in Hungarian). Orv Hetil.

[6] Louthrenoo W, Trongkamolthum T, Kasitanon N, Wongthanee A (2021). Predicting factors of adverse pregnancy outcomes in Thai patients with systemic lupus erythematosus: a STROBE-compliant study. Medicine (Baltimore).

[7] Kharbanda R, Naveen R, Misra DP, Gupta L, Agarwal V (2021). Poor maternal and foetal outcomes in women with systemic sclerosis: an interview-based study at a tertiary centre. Rheumatol Int.

[8] Götestam Skorpen C, Lydersen S, Gilboe IM (2017). Disease activity during pregnancy and the first year postpartum in women with systemic lupus erythematosus. Arthritis Care Res (Hoboken).

[9] Skinner-Taylor CM, Perez-Barbosa L, Barriga-Maldonado ES (2021). Reproductive health counseling and contraceptive use in Mexican women with rheumatic diseases: a cross-sectional study. Rheumatol Int.

[10] Molad Y, Borkowski T, Monselise A (2005). Maternal and fetal outcome of lupus pregnancy: a prospective study of 29 pregnancies. Lupus.

[11] Mankee A, Petri M, Magder LS (2015). Lupus anticoagulant, disease activity and low complement in the first trimester are predictive of pregnancy loss. Lupus Sci Med.

[12] Tektonidou MG, Andreoli L, Limper M, Tincani A, Ward MM (2019). Management of thrombotic and obstetric antiphospholipid syndrome: a systematic literature review informing the EULAR recommendations for the management of antiphospholipid syndrome in adults. RMD Open.

[13] Redman CW, Sargent IL (2009). Placental stress and pre-eclampsia: a revised view. Placenta.

[14] Pijnenborg R, Vercruysse L, Hanssens M (2006). The uterine spiral arteries in human pregnancy: facts and controversies. Placenta.

[15] Girardi G, Redecha P, Salmon JE (2004). Heparin prevents antiphospholipid antibody-induced fetal loss by inhibiting complement activation. Nat Med.

[16] Albrecht ED, Aberdeen GW, Pepe GJ (2000). The role of estrogen in the maintenance of primate pregnancy. Am J Obstet Gynecol.

[17] Lee EE, Jun JK, Lee EB (2021). Management of women with antiphospholipid antibodies or antiphospholipid syndrome during pregnancy. J Korean Med Sci.

